# “Ten Days of Paid Incarceration and Mental Torture” Experiences of Quarantined Individuals Arriving in the UK from Red Listed Countries in Southern Africa Amid the COVID-19 Pandemic

**DOI:** 10.1007/s40615-023-01574-w

**Published:** 2023-03-27

**Authors:** Mathew Nyashanu, Michael Brown, Ticahaenzana Nyashanu, Diana Frost, Fungisai Mushawa

**Affiliations:** 1 Nottingham Trent University, 50 Shakespeare Street, Nottingham, NG1 4FQ UK; 2 Department of Psychology, Faculty of Humanities, University of Pretoria, P Bag X20, Hatfield, Pretoria, South Africa

**Keywords:** Mental health, Quarantine, Health outcomes, COVID-19 pandemic, Silence framework

## Abstract

COVID-19 has affected many communities across the world prompting different strategies of containing it. The strategies to contain COVID-19 included restrictive environments such as self-isolation and quarantine. This research study was set to explore the experiences of quarantined individuals arriving in the United Kingdom (UK) from red listed countries in Southern Africa. This research study utilises an exploratory qualitative approach. Semi-structured interviews were used to collect data from twenty-five research participants. A thematic approach underpinning the four phases of data analysis in The Silence Framework (TSF) was used to analyse the data. The study found that the research participants reported confinement, dehumanisation, feeling swindled, depressed, anxious and stigmatised. Less restrictive and non-oppressive quarantine regimes should be considered to foster positive mental health outcomes on individuals undergoing quarantine during pandemics.

## Background

The COVID-19 pandemic has affected the global community, with the impact felt across all age groups [1]. In a bid to curb the spread of the disease, restrictive environments such as self-isolation and quarantine were put in place. Self-isolation typically describes individuals who either have travelled through a region with cases of COVID-19 or are suspected to have COVID-19, and are required to remain at their residential property for a set period of separation [2]. Similarly, quarantine is the separation of those individuals presumed to be healthy but have been in contact with suspected or positive COVID-19 cases [3], where such separation normally takes place at a managed place, hospital or hotel. The United Kingdom (UK) first instituted self-isolation in February 2020 [3] and instituted managed quarantine in 2021 following the discovery of the delta variant. To further contain the spread of coronavirus, the UK government placed some countries on a red list, such that passengers travelling from these countries faced additional quarantine measures. Most of the Southern African countries—including Zimbabwe, South Africa, Botswana, Namibia, Lesotho and Eswatini—were considered red list countries. Individuals travelling from these countries were therefore placed in managed quarantine on entry to the UK. This detention of these individuals in managed quarantine facilities has been criticised for contributing to a wide spectrum of socio-psychological distress and physical health challenges, as well as negative economic consequences [1].

There is however a paucity of research on the negative consequences faced by detained persons [1], and the exact nature and extent of these perceived negative consequences. In managed quarantine centres and other restrictive environments, children are particularly at an elevated risk of developing physical and mental health problems [4, 5] as the isolative and restrictive nature of the hotel-based detention facilities may compromise children’s access to nutritional necessities and essential health services [4, 6]. Furthermore, children’s engagement in play and recreational activities enhances expressiveness, improves social connectivity and promotes skills [7]. In the constrictive environments such as quarantine centres, however, children are limited in physical and social space, thereby hindering their physical and psychological development [8]. The longer or the more frequent the children are confined in these spaces, the more they are likely to present deficiencies in certain skills and attributes.

Quarantine measures also impact on the adult population group, with additional negative implications such as economic strain. The cost of staying in a detention centre is considered exorbitant for many people in the low-medium income bracket. For example, the UK’s Corporate Travel Management (CTM) administered the bookings for managed quarantine facilities for travellers from red list countries at a rate of £2285 for a single adult, with an additional rate of £1430 for one adult or a child over 11 years of age. Furthermore, most individuals are likely to incur loss of income and experience other financial burdens whilst in detention centres, causing significant socio-economic distress.

This study therefore explores the experiences of quarantined individuals from red listed countries. Many studies have concentrated on the positive aspects of quarantine in public health without a close analysis of the individuals who are quarantined. This study endeavours to feel this gap by exploring the experiences of individuals who have undergone quarantine. The quarantine facilities were established in the interest of public safety; however, the restrictive nature of the facilities and the implementation of certain quarantine procedures raised human rights and ethical concerns from many organisations [9, 10]. Individuals faced limited freedom of movement, disruption of their daily routines and little access to health, social and other essential services. There were also dietary concerns as the quarantine centres offered basic, generalised diets that did not necessarily cater for specific needs of individuals such as dietary requirements for health conditions [11] or religious or cultural purposes. Ethical concerns were therefore raised relating to the rights of quarantined individuals with respect to medical care, freedom of movement and access to nutrition based on diverse cultural backgrounds [9, 11, 12].

Comparative studies in Australia and New Zealand suggest that shared spaces (such as hotel-based quarantine facilities) are less effective when compared to purpose-built facilities [13] as the latter typically have better ventilation and therefore reduced risk of transmission of the virus[12]. In shared spaces (particularly those operating beyond their limit), hygienic practices may be compromised, thereby exposing detained individuals to further health hazards—a particular concern for individuals faced with pre-existing health conditions. Lockdowns and confinement of individuals in quarantine facilities therefore contribute to psychological distress in the form of frustration, anxiety and loneliness [14]. In a related study in Western Nepal involving individuals in COVID-19 quarantine facilities, results showed significant levels of anxiety and depression [9, 12]. According to the authors, people develop a constant fear of contracting the virus within the shared space. This fear is transferred to others with whom they interact after leaving the quarantine facility, and thus, persons leaving managed facilities experience stigmatisation and discrimination at workplace, school and other places in society [15, 16].

This study therefore examines the experiences of individuals from red list countries who have been detained in managed quarantined facilities (specifically hotels) in the UK. The research examines the discrimination faced by these detained individuals, and the implications for their long-term mental health outcomes.

## Methodology

This research study utilised an exploratory qualitative approach to explore the experiences of quarantined individuals from red listed countries in Southern Africa [17]. The intention of using an explorative qualitative approach was to better understand the topic as opposed to offering a final and conclusive solution to the matter under investigation [18]. The method has the potential to identify possible areas for further investigations into the subject and provide an overview of the issue under investigation from a new perspective, leading to key information for future interventions [19].

### Data Collection and Recruitment

The data was collected using individual semi-structured interviews to explore the experiences of individuals from red listed countries in Southern Africa who had been quarantined under COVID-19 in UK hotels. The interview guide was designed and informed by the literature from previous primary and secondary research studies on COVID-19 and other pandemics amongst communities [20]. Prior to the interviews, a pilot study involving four (4) research participants was carried out to test the feasibility of the interview protocol on the experiences of quarantined individuals. The four (4) interviews were conducted via an online platform called MS Teams. The use of Teams online platform as a method of facilitating data collection was meant to be in line with the notion of social distancing enforced by the central government to curtail the spread of the COVID-19 pandemic in the UK.

After piloting the interviews, the four (4) research participants were invited to comment on the suitability of the interview schedule with regard to understanding and responding to the questions. The four (4) research participants did not suggest any changes to the original interview schedule as they felt that it was clear and suitable for the research study in question. Twenty-five (25) individual semi-structured interviews were held with individuals who had been quarantined for 10 days in a UK government managed hotel. All the research participants had travelled to the UK from Southern Africa. The researchers sent letters and information sheets to religious organisations and Southern African community groups in the UK inviting their members to take part in the research study. Only those who had agreed to take part in the research study had their names and telephone contacts forwarded to the researchers by the leaders of religious organisations and community groups to organise interview dates and time. The interviews were held through Teams. As alluded to earlier on, the use of Teams, an online platform, was due to adherence to social distance protocols meant to prevent the spread of COVID -19 as the interviews took place during the COVID 19 pandemic period. Prior to the interviews, research participants were given an opportunity to read and understand the information sheet before asking questions for any clarifications. Furthermore, all the twenty-five (25) research participants signed a consent form, which granted them the right to withdraw from the study at any time without giving reasons. They were also assured that their withdrawal from the study will not impact on their present provision of any service or right. The interviews lasted for approximately 30 min and were conducted in English. Table 1 shows the semi-structured questions that were used as the bases of the 25 interviews with the research participants.

**Table 1:** Semi-structured questionnaires for research participants

1. Why did you travel to Southern Africa during the COVID-19 pandemic?
2. Please can you tell me what happened from the time you landed at the airport in the UK until you were taken to the hotel?
3. Please can you describe your feelings during the 10 days you spend at the hotel?
4. Can you describe how you were treated and your feelings during the 10 days that you spent at the hotel?
5. Please can you describe your feelings when you were asked to pay an amount of £2285.00 for your stay at the hotel?
6. What are the positive things that you took from your experience of undergoing quarantine?
7. What would you want to be improved when the government conduct quarantine in future pandemics?

### Inclusion and Exclusion Criteria

The inclusion criteria included men and women who had travelled to red listed countries in Southern Africa and quarantined in government-managed hotels upon return to the UK [21]. All the participants had spent 10 days quarantined in a government-managed hotel. It was important to recruit a heterogeneous sample with respect to where they came from (that is Southern Africa) so as to elicit information from people who were from the same region and affected by the same environmental factors.

### Ethical Considerations

The research was vetted and approved by the Nottingham Trent University ethics committee. Research participants were given information sheets to read and understand the information concerning the study. They also signed consent forms which gave them the right to withdraw from the study without giving any reason.

## Data Analysis

**Fig. 1:**
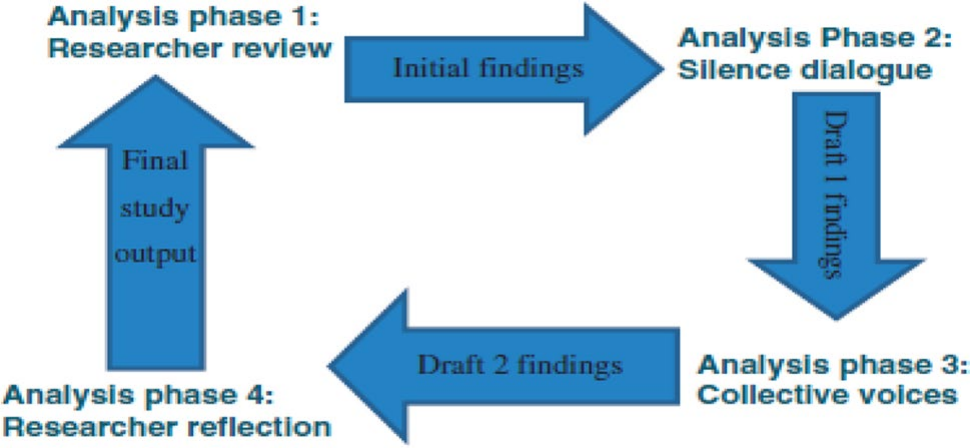
The four phases of data analysis

All interviews were video-recorded through the online platform and transcribed verbatim. NVivo was used to organise the data and make it easy for analysis. For the verification of accuracy, all transcriptions were read back to the research participants for confirmation of the main points through virtually on Teams. A thematic approach underpinning the four phases of data analysis in The Silence Framework (TSF) was utilised [22]. See Fig. 1 showing the four phases of TSF.

In the first instance, the researchers read the transcripts repeatedly to identify and ascertain the accounts of experiences that were important to the research participants. The themes identified were supported by captions from research participants. This formed the bases of the first draft of themes. In the second phase, the researchers took the draft to the research participants for verification of the themes and captions used to support them. Following confirmation of the themes, the draft from phase 2 was taken to the user voice group in phase 3; this was a group of people who had been quarantined in a government-managed hotel but had not taken part in the research study. The idea was to subject the findings to a critical associative eye [22]. Following confirmation of the first draft in phase 3, the data was taken to phase 4 where the researchers analysed the output from phase 3 to form the final output of the research study which were presented as the main findings.

## Results

The study found that the research participants reported confinement, dehumanisation, feeling swindled, depressed, anxious and stigmatised.

### Confinement

All the twenty-five (25) research participants reported that they were confined in a room sometimes alone or with a child. Some research participants drew comparisons that likened their situation at the time to solitary confinement typical of the modern-day prison.

I spent most of the time in a room alone looking at the walls and feeling like a prisoner in solitary confinement……..I was only allowed 30 minutes outside per day that’s 5 hours in ten days…..Honestly, I don’t wish anyone to go through such horrendous experience. *A 40-year-old man who had travelled to attend a brother’s funeral*

I was breast feeding spending all day sitting or lying on the bed my child felt distressed as I could not stay outside for a long time…….This plan was not well thought out I will live with this scar in my life forever……..I felt like a prisoner of war being tortured**.** *A 30-year-old women who had travelled to see her sick father*

### Dehumanisation

Nearly all the research participants, twenty-four (24), reported feelings of being dehumanised by being allowed only 30 min to go out under close monitoring. They also reported being treated as though they had COVID-19 even though they had tested negative prior to boarding the plane.

It was shocking to see hotel staff leaving my food outside the door and quickly walk away as though I had contracted a dangerous disease, yet I had no COVID-19…….The way hotel staff were shouting at me like I was a prisoner of some sort…...I have never experienced such disrespect in my life. *A 30-year-old woman who had travelled to see a sick relative*

I can’t imagine that I was only allowed 5 hours to see the sun in ten days……I committed no crime only that I had gone to see my sister who was not feeling well…….I felt that all my rights as a human being were withdrawn for ten days**.** *A 45-year-old man who had travelled to see a sick sister*

### Swindled

All the twenty-five (25) research participants reported that COVID-19 quarantine facilities for travellers from red list countries were charged at a rate of £2285 for a single adult, with an additional rate of £1430 for the second adult or a child over 11 years of age. They felt that it was like they were being swindled of their hard-earned money.

I have stayed and worked in the UK for more than 25 years never in my life I have seen people being asked to pay such colossal amounts of money within a day ……..completely without a notice…….This is criminal where will I get such an amount of money within such a short time. *A 50-year-old man coming from a relative funeral*

This is purely a swindling game I think some other people were thinking of making money during COVID-19 and thought that it’s easy to swindle it through people from Southern Africa……..The British Prime minister was quick to put the whole of Southern Africa on red list, yet this omicron virus was all over the world. *A 45-year-old woman coming from seeing a sick relative*

### Depressed

All the twenty-five (25) research participants reported getting depressed during the 10 days that they were living in quarantine. They likened the quarantine regime to a penitentiary centre. Furthermore, they reported poor mental health support during the quarantine period.

I felt so depressed nearly every day that I was in the quarantine……………It was like I was in a penitentiary centre with no mental health support despite the poor and depressing conditions that I was living**.** *A 40-year-old woman coming from visiting a family after 10 years*

The place was just like a mini penitentiary full of depressed people……… I had no one to talk to spending most of my time looking at the four walls of my room….. I have still not recovered from the impact of the ten days in quarantine.* A 35-year-old man coming from attending a relative funeral*

### Anxiety

Nearly all the research participants, twenty-four (24), reported feelings of high anxiety as they feared to contract COVID-19 whilst in quarantine. Furthermore, they reported anxiety due to fear of living alone for the next 10 days.

I could not imagine that I was going to spent 10 days in a room alone…..I had never done this in my entire life I was anxious and fearful of what I was going to go through in the coming ten days. *A 25-year-old man coming from seeing a sick mother*

I felt so anxious I could not imagine that that I was going to spent ten days in a lonely room……..More so, I was feeling so anxious when we were being transported to the hotel as we were put together with other people from different flights prompting the possibility on COVID-19 infection. *A 29-year-old man coming from a funeral*

### Stigmatised

All the twenty-five (25) research participants reported being exposed to high stigma by both staff escorting them to the hotel and those taking control of the hotel. They reported possibility of misinformation about their COVID-19 status at the time of checking into the hotel**.**

I was so unhappy with the way we were I was being treated the people taking us to the hotel treated us as though we had COVID-19 yet we were tested before boarding the plane and our results were negative……..There was no way we would have gone of the plane with a positive test. *A 43-year-old man coming from seeing a sick father*

The staff taking us to the hotel had already labelled us COVID-19 positive by the way they were looking and talking to us……..It was even worse at the hotel the staff there seems ill informed about our status I think most of them were thinking that we had COVID-19 their actions were highly stigmatising. *A 29-year-old woman coming from attending a relative funeral*

## Discussion

Confinement in a room alone for a long time can cause feelings of loneliness and sometimes anxiety [23]. It can also trigger stress and negative feelings resulting in poor health outcomes. In this study, the research participants reported that they were confined in a room sometimes alone or with a child. Some research participants drew comparisons that likened their situation at the time to solitary confinement typical of the modern-day prison. Considering the above findings, it is important for governments to consider the well-being of people when putting them in quarantine through the use of supportive and interactive methods like allowing them to be visited by relatives following their first negative PCR test on the second day of quarantine. The visiting relatives can also be asked to take a COVID-19 PCR test prior to their visit to ascertain that they are negative. Such an arrangement can help individuals in quarantine to cope with periods of confinement in the room.

Feelings of dehumanisation because of having or suspected to have a condition can trigger stressful moments and negativity [24]. Such feelings have been evident in situations where people have been dehumanised because of their condition, for example with HIV and leprose [25]. In this study, the research participants reported feelings of being dehumanised by being allowed only 30 min to go out under close monitoring. They also reported being treated as though they had COVID-19 even though they had tested negative prior to boarding the plane and on the second day of checking in the hotel. In light of the above finding, it is important that staff receiving and manning the quarantine spaces need to be trained on how to handle and support individuals coming into quarantine to avoid inhumane treatment and subsequent stressful moments. It is also important for the staff to know that not all the people in quarantine will have tested positive for COVID-19; as such, normal COVID-19 precautions should be applied when dealing with them as opposed to disrespectful treatment as reported by the research participants. Therapeutic services could be offered at these quarantine hotels to support the emotional well-being of people under detention particularly in view of the high rates being paid to stay in these hotels.

Paying money for something that individuals may perceive is not worth the price can trigger feelings of being swindled [26]. More so, it can be worse if the amount of money to be paid is remarkably high and likely to put them in debt. In this study, the research participants reported that they felt that it was like they were being swindled of their hard-earned money. Considering the above finding, it is important that quarantine costs are covered by the central government given the unfavourable economic situations that were brought by the COVID-19 pandemic which included amongst other things individuals losing their jobs. Such an assistance can help individuals to stay out of debt during grim times and avoid negative impact on their health and well-being. It can be argued that these people chose to travel when there were restrictions in place due to the pandemic; however, it is worth noting that due to cultural differences between the UK and Southern Africa, expectations of family members where there is bereavement and death differ, with Southern African countries being close knit communities.

Feelings of depression can have a negative impact on the health and well-being of an individual [27]. In other cases, it can take longer for people to recover following a bout of depression [28]. This can in turn have implications on the total health costs of the government given the expenses associated with caring for people affected by mental health. In this study, the research participants reported getting depressed during the 10 days that they were living in quarantine. They likened the quarantine regime to a penitentiary centre. Furthermore, they reported poor mental health support during the quarantine period. It is therefore important that the government consider other methods of quarantine which are not stressful for the individuals being quarantined; for example, individuals can be released to quarantine at home following a negative PCR test on the second day of quarantine since a negative PCR test is a confirmation that an individual does not have the COVID-19 virus. This can save a lot of money on the part of the individuals who were being asked to pay colossal sums of money for a 10-day quarantine period. More importantly, it would have prevented feelings of depression for many people entering quarantine.

Anxiety can be associated with feelings of fear and the unknown [29]. It can be triggered through negative experiences impacting on the health and well-being of an individual [30]. In this study, the research participants reported feelings of high anxiety as they feared contracting COVID-19 whilst in quarantine. Furthermore, they reported anxiety due to fear of living alone for the next 10 days. In light of the above finding, quarantine centres need to have qualified mental health practitioners ready to help individuals who may be anxious about getting into quarantine.

Stigma can trigger feelings of being discriminated whether it is internalised or real [31]. Feelings of being discriminated against can result in people feeling treated unfairly and unequally. It has been noted that the effect of lockdown has been deeply unequal in the British society, reinforcing and exacerbating existing divides [32]. Such feelings can impact on the health and well-being of the affected individuals as seen in those exposed to high stigma in communities following HIV infection [33]. In this study, the research participants reported being exposed to high stigma by both staff escorting them to the hotels and those staffing the hotels. They reported possibility of misinformation about their COVID-19 status at the time of checking into the hotel considering the treatment they received from the hotel staff. It is therefore important that staff escorting individuals and manning the hotels need to be trained on stigma awareness and prevention. This will enable them to treat the individuals they are supporting with dignity and respect [34].

### Limitations of the Study

The research study was conducted with travellers from Southern Africa; however, research involving travellers from other regions on the red listed countries may be needed in the future to enable comparisons of experiences across regions. This research was also qualitative in nature; future research could use a mixed-methods approach to enable broader exploration of the issues from different ontological and epistemological positions. Research participants were recruited through community groups privy to the researchers and this may have caused a bias effect; in the future, opening the recruitment base of research participants to the wider community would mitigate the bias**.**

### Implications for Practice

There is a need to consider the health and well-being of all people coming into quarantine to ensure that their health outcomes are positive following the experience. It is also important that governments should opt for less restrictive and non-oppressive quarantine regimes to enhance treatment of individuals with respect and dignity. However, it is also important to note that when there is a pandemic like COVID-19 the government has an obligation to protect vulnerable people which will automatically support the case for quarantine. In deciding which countries should be quarantined, governments and political leaders need to be informed by objective public health evidence galvanised by the spirit of social justice as opposed to decisions based on political alliance or opposition.

## Concluding Comments

It is important that all individuals coming into quarantine are supported with respect and dignity. More importantly, there is a need to consider scientific evidence in making decisions that are health related like quarantine as opposed to political motives and positions. Less restrictive and non-oppressive quarantine regimes should be considered to foster positive mental health outcomes on the affected individuals. There is a need for further research to develop evidence-informed implementation strategies that minimise creating negative outcomes in the process of trying to curb the spread of the virus. There is lack of clarity of purpose and a transparent operational plan to adequately validate hotel-based quarantine approaches as the best practice to mitigate the risks of spreading of COVID-19. This has led to some critics labelling the whole exercise of red listing and quarantine as a politicised, monetarised and racial scheme [35, 36]

## Data Availability

Data is available on request provided the requesting part has a data management plan that safeguards the data.
